# Seasonal characteristics and key sources of trace element deposition fluxes in coastal Poland

**DOI:** 10.1038/s41598-025-32170-z

**Published:** 2026-01-05

**Authors:** Patrycja Bukowska

**Affiliations:** Institute of Meteorology and Water Management, Waszyngtona 42, Gdynia, 80-342 PL Poland

**Keywords:** Climate sciences, Environmental sciences

## Abstract

Coastal regions are important sources of anthropogenic elements that contribute to climatic, hydrological, and environmental changes. The Baltic Sea basin represents a key domain for exploring air quality issues, where inter-annual variability of various pollutants is notable. Trace element atmospheric dry deposition fluxes were investigated for the first time at a coastal urban site in Poland between April 2019 and May 2020. Atmospheric deposits of As, Cd, Co, Cr, Cu, Fe, Mn, Mo, Ni, Pb, Sb, and V were calculated using the deposition velocities for coarse-mode particles (PM_10_), considering different meteorological parameters. Data analysis revealed that Ni and As contributed substantially to more than 30% in winter, indicating a significant impact of specific anthropogenic sources (i.e., industrial combustion and coal combustion for domestic heating). Cr accounted for 19%-33% of the total deposition flux, with its highest contribution in spring. The principal component analysis revealed that primary sources, including road- and soil-related dust resuspension, vehicle exhausts, coal combustion, and the industrial sector, had a substantial influence on the total variation in deposition fluxes. The HYSPLIT analysis identified regions such as the northwestern and western Europe as significant for enhancing atmospheric deposition of trace elements in coastal Poland.

**Supplementary Information:**

The online version contains supplementary material available at 10.1038/s41598-025-32170-z.

https://orcid.org/0000-0002-3928-3031.

## Introduction

Atmospheric particulate matter (PM) has a significant impact on weather and climate changes, the radiative budget, atmospheric processes, air quality, human health, and ecosystems. Coarse-mode particles, smaller than or equal to 10 microns (PM_10_), are crucial to human-influenced negative forcing on climate. Atmospheric deposition is critical for understanding trace element (TE) balance and their contribution to air quality and regional/global climate^1–4^. Multiple field studies of trace element abundance in the PM have focused on various compounds, including Mn, Zn, Cu, As, Pb, Fe, and some elements considered carcinogenic, such as Cd, As, Cr, and Ni. Many studies in Europe^5–9^, the United States^10,11^, Asia^12,13^, and South America^14^ have widely discussed the PM_10_-bound trace element concentrations, seasonal variation, and their anthropogenic emission sources. However, deposition trends are available only in limited studies^15–18^. Measurements related to deposition fluxes are a key step in understanding interactions between air quality, human health, and the ecosystem; thus, there is still a longstanding concern about providing robust data to models or deep-learning approaches to predict trends in atmospheric loading of pollutants. Deposited trace elements originating from industrial activities (i.e., mining, refineries, and smelting) can lead to degradation of the soil system^19^.

Up to now, the atmospheric deposition measurements in the Baltic Sea countries are still very limited, and thus, coastal regions need to improve data on the chemical composition of size-fractionated PM. This is especially important regarding metallic species that have inherent cellular toxic properties and can exhibit remarkable bioaccumulation in the human body, leading to health damage, diseases (e.g., lung, bloodstream, nerve, and cardiovascular systems), and cancers. In addition, the chemical profile of PM is insufficiently discussed for many inland anthropogenic source categories (i.e., refinery and shipping activities) in coastal Poland. The contribution of regional emission sources to air quality and their potential impact on the coastal ecosystem remains scarcely quantified. Unlike previous preliminary studies^20^, which focused on trace element deposition and considered only the PM_2.5_ fraction, the present study aims to fill gaps in our understanding of the current status of atmospheric deposition of trace element-containing coarse-mode particles, using meteorological data and backward trajectory simulations from the NOAA HYSPLIT model.

The central scientific point of this research was to quantify and characterize the variability of trace element deposition fluxes in different seasons and to determine the contribution of various anthropogenic emission sources. The present study analyzes 344 filter samples collected in all seasons. It provides the full characteristics of As, Cd, Co, Cr, Cu, Fe, Mn, Mo, Ni, Pb, Sb, and V over the urban coastal region in northern Poland. To date, no comprehensive studies have been conducted on the deposition of PM_10_ in the coastal areas of Poland. The specific objectives and novelties of this study were as follows:

1. to conduct principal component analysis on the source apportionment of trace elements deposition;
2. to establish the main meteorological drivers of changes in the intraseasonal variability of trace element dry deposition;
3. to investigate the relationships between dry deposition fluxes and wind clusters from the HYSPLIT model.

## Methodology

### Site description

**Fig. 1:**
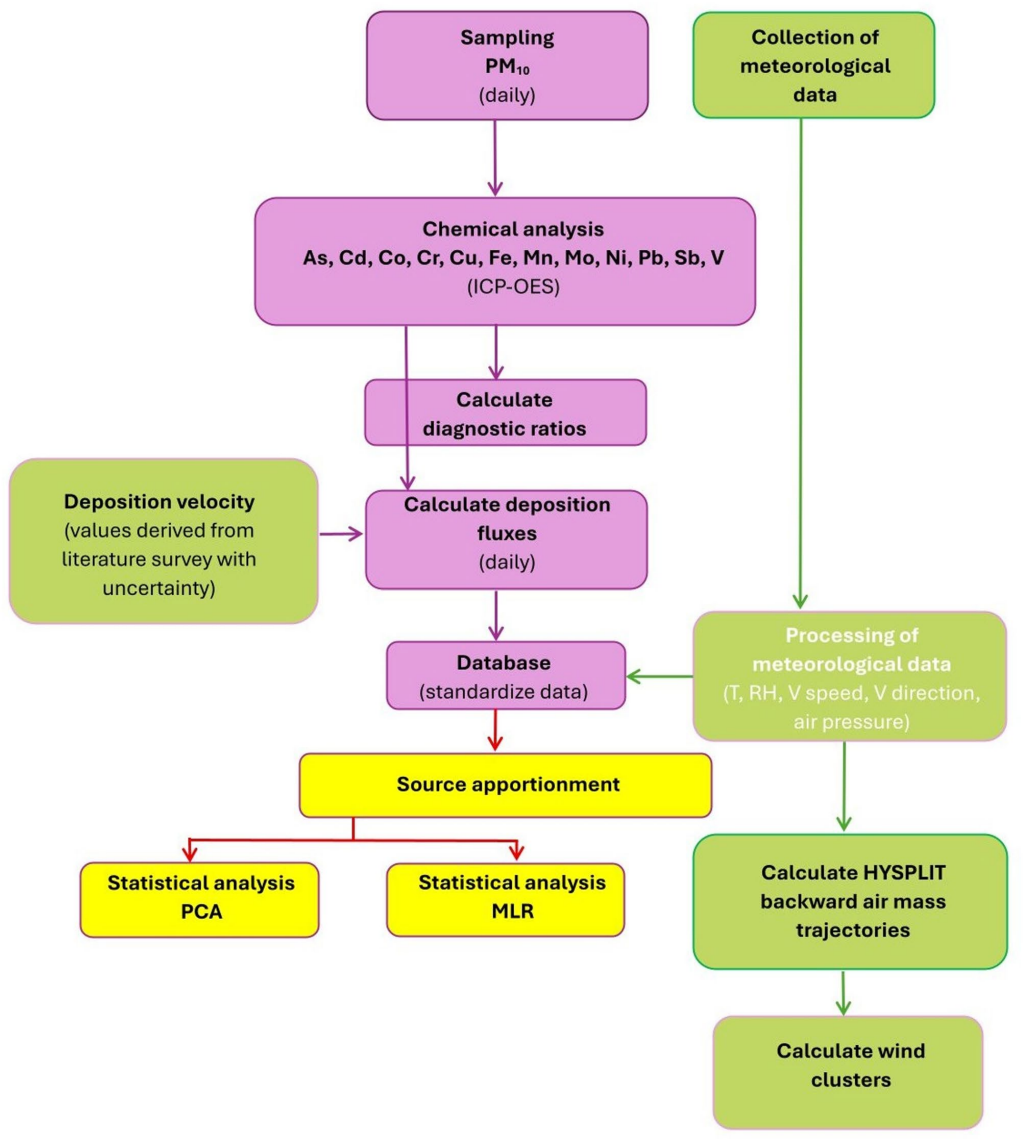
Flowchart of the methodology developed in this study.

 In this work, daily PM_10_ samples were collected from the Gdynia site (54 °52’55.1″N, 18 °56’13.5″E), representing the coastal urban region in northern Poland, from April 2019 to May 2020. A low-volume (2.30 m^3^ h^− 1^ EU 12341) manual Comde Derenda system equipped with quartz fiber filters (QMA, Whatman, 47 mm diameter) was used for sampling. This measurement system was deployed on the rooftop of a 4-store building at a height of ca. 20 m above ground level. The methodology employed in this study is presented in Fig. 1. The sampling site is influenced by major anthropogenic factors, including traffic, shipping, domestic heating, industrial activities, the heavy petrochemical industry, and primary and secondary anthropogenic aerosols (Fig. S1).

### Experimental setup

 Before sampling, the filters were pre-heated at 500 °C for 8 h and then conditioned for 24 h in a borosilicate glass desiccator at a constant temperature of 24 °C and a humidity of 40%. They were then weighed on a microbalance with a precision of 10^− 6^ g. The total number of PM_10_ samples collected between April 2019 and May 2020 was 344. After sampling, filters were again conditioned for 24 h, weighed, and then frozen at -20 °C in aluminum foil and a zipped bag until chemical analysis, as shown in Siudek^21^. All handling work with filters was done in a clean room facility with ISO standard (HEPA units), while quality control was routinely achieved following weekly validation procedures. In the present study, the PM_10_ mass concentration (µg m^− 3^) during the sampling period from April 2019 to May 2020, encompassing the period before and at the onset of the COVID-19 pandemic, ranged from 3.4 to 65.5, with a mean value of 17.7 ± 10.5. This finding indicates that coarse-mode particles exceeded the annual mean World Health Organization value (15 µg m^− 3^), and 1.46% of the daily data (5 days in total) showed values greater than the WHO limit for 24-h PM_10_ standards (45 µg m^− 3^).

### Meteorological data

To analyze the meteorology-driven variability of trace element dry deposition, a standard dataset was used, including air temperature (T, °C), air pressure (p, hPa), relative humidity (RH, %), wind speed (WS, m s^− 1^), and wind direction (Fig. 1). Meteorological variables were initialized at the site from forecasting and air quality sensors (Comde Derenda) at 1-h intervals during the field campaign and used to assess the impact of weather factors on the daily, monthly, and seasonal mean PM_10_ deposition profile and transport patterns. Data were averaged to a 24 h resolution to match the PM measurements. The lowest 24-h mean air temperature was observed in December (TMIN=-0.5 °C), while the highest 24-h mean was in June (TMAX = 26.2 °C). During the sampling period, the mean relative humidity (RH) was 74%, with peaks in January and a minimum in June. Details can be found in Table S1 (supplementary information), presenting a summary of meteorological data (i.e., mean air temperature, mean air pressure, mean relative humidity, and mean wind velocity) during the PM_10_ measurements. The predominant wind direction during winter was from the south, whereas strong west and southwest winds were more frequent for spring and summer measurements. The meteorological seasons were as follows: spring (March-May), summer (June-August), autumn (September-November), and winter (December-February).

### The HYSPLIT model

 This study utilizes the National Oceanic and Atmospheric Administration (NOAA) Hybrid Single Particle Lagrangian Integrated Trajectory Model (HYSPLIT)^22^ to visualize the origins of air masses and their transport patterns from April 2019 to May 2020. A series of 344 two-day Lagrangian backward trajectories were calculated using initial data from the global data assimilation system (GDAS), including a temporal resolution of 6 h, a spatial resolution of 1°, a starting point at a height of 20 m a.g.l., and altitudes of 500, 1000, and 1500 m.

The HYSPLIT analysis of backward trajectory simulations was employed to investigate the circulation patterns within the study domain. To facilitate the analysis of potential sources of trace element deposition and the evolution of air masses before they reach the study domain, each trajectory is assigned to one wind cluster, representing their prevailing circulation patterns i.e., north (N), northeast (NE), east (E), southeast (SE), south (S), southwest (SW), west (W), northwest (NW), and local cluster (L). For statistical analysis of differences between wind clusters, the Kruskal-Wallis test was applied. The target region for this study is illustrated in Fig. S1. Details on the contribution of air mass clusters to the transport pathway over the study domain, along with the characteristics of air masses arriving at the coastal city of Gdynia, are provided in Table S2 in the Appendix. The results are discussed in Section: *Importance of wind clusters*.

### Chemical analysis

In this study, the concentrations of 12 elements, including As, Cd, Co, Cr, Cu, Fe, Mn, Mo, Ni, Pb, Sb, and V in PM_10_ filter samples were determined by using an inductively coupled plasma-optical emission spectrometer (ICP-OES, Varian, Agilent). The ISO standard for determining metals in PM samples using ICP-OES is ISO 15202-2:2020. Quality assurance and quality control (QA/QC) measures were implemented throughout the sampling period. The detection limit (µg L^− 1^), calculated as three times the standard deviation of reagent blanks for each element, was as follows: As (3.75), Cd (0.40), Co (0.71), Cr (0.65), Cu (0.89), Fe (4.64), Mn (0.06), Mo (0.94), Ni (0.69), Pb (3.35), Sb (1.09), and V (0.90). The limit of quantification (LOQ) for 12 elements ranged between 0.18 (Mn) and 13.94 (Fe). Trace elements were analyzed from a quarter of quartz membrane filter samples, which were digested in a closed Teflon vial with 5 mL of 65% Suprapure nitric acid solution (Merck, Germany) for 20 min at 170 °C using a microwave digestion system (MARS5, CEM, UK). After this, the digested solution was placed on pre-cleaned polypropylene Falcon tubes and filled to 13 mL with high-purity double-deionized water. The recovery of amounts of trace elements from standard reference materials (BCR-176R Fly Ash and ERM-CZ120 Fine Dust) was estimated under the same analytical procedure. The recovery rates of As, Cd, Co, Cr, Cu, Fe, Ni, and Pb in PM_10_ reference samples were 99%, 106%, 86%, 104%, 95%, 93%, 89%, and 91%, respectively. The procedure for chemical identification of trace elements using ICP-AES and pre-treatment with HNO_3_ digestion is consistent with Becagli et al.^23^.

### Statistical analysis

In this study, data analysis was performed using TIBCO Statistica version 13.3. The Shapiro–Wilk test (*p* < 0.05) was used to evaluate the normality of the data, and a nonparametric Kruskal–Wallis test was used to examine the seasonal variation in deposition fluxes of elements, also testing the effect of seasons. The Spearman correlation coefficient and hierarchical cluster analysis were employed to investigate the relationships between variables, and multiple linear regression (MLR) was used to model the combined meteorological effects on PM. Basic descriptive statistics (mean, median, standard deviation, minimum, and maximum values) were calculated for each sampling month. Statistically significant differences were determined at a 95% confidence level, and *p*-values were considered at *p* < 0.05. The present study employs principal component analysis (PCA), specifically the number of eigenvalues greater than 1 (Costabile et al.^24^), under varimax normalized rotation and the Kaiser-Meyer-Olkin criterion, as well as Bartlett’s test, to examine the adequacy and sphericity of the measured dataset. Moreover, a summary of PM_10_ mass concentration and deposition fluxes (DFs) for each trace element was presented in Table S3.

### Dry deposition flux estimation

 To estimate the particle fluxes of target elements during the sampling period, a simple model is fitted for each PM component:

1 $$DF = C \times Vd$$

*DF* represents the daily deposition flux of individual elemental species with PM_10_ in µg m^− 2^ day^− 1^. *C* denotes the concentration of each element in coarse-mode particles in ng m^− 3^; and *Vd* is the deposition velocity of PM_10_ in cm s^− 1^. The constant factors of 86,400 and 10^− 2^ were used to convert the units from seconds to days and cm to m, respectively.

Due to the lack of observational data on dry deposition rates of particles within the studied area, the total depositional fluxes are estimated using an inferential method. *DF* results are interpreted using velocity values derived from a literature survey^1,9,25,26^. Specifically, it is assumed that the *Vd* is attributable to 4.2 (Cr, Mn, Pb)^1^, 2.0 (Fe)^25^, 1.8 (Cu, Mo)^1^, 0.52 (Ni, V)^*1*^, and 0.5 (As, Cd, Co, Sb)^25^ cm s^− 1^, accordingly to the average deposition velocity for these elements. In addition, *Vd* values are assumed to be due to gravitational settling, following the analytical method described by Fan et al.^27^. It should be noted that the deposition velocity is sensitive to fluctuations in wind speed, relative humidity, and particle size^28^. Several studies have highlighted that uncertainties in dry deposition measurements or estimates, or based on an empirical algorithm, can be up to a factor of 2 or larger^29,30^. *DF* results in this study were based on an analogy to previous coastal studies^16^; however, they represent the Baltic coastal climatic conditions.

## Results and discussion

### Comparison with previous studies

Depositional fluxes of trace elements calculated for campaigns in the spring-winter of 2019 (expressed as a sum) are shown in Table 1. Results were compared to those of other worldwide regions to show the distribution pattern of trace elements deposition in Poland in the context of global TEs deposition levels.

**Table 1:** Summary of the annual dry deposition fluxes of elements. Values are in µg m^− 2^ year^− 1^.

Trace element											
	Tasdemir and Kural^33^	Zhang et al.^34^	Desboeufs et al.^16^	Sakata et al.^32^	Gray et al.^31^	Chen et al.^17^	Shi et al.^15^			Fu et al.^18^	Present study
**As**	–	–	60				1703	2092	2897	210	397
**Cd**	1100	–	–	390	20	600	52	88	137	50	37
**Co**	2920	–	–	–	–	–	–	–	–	–	30
**Cr**	22 260	9200	50	6200	2800	6000	–	–	–	120	1008
**Cu**	71 170	20 800	330	16 000	3500	11 000	–	–	–	590	606
**Fe**	29 230	1520	10 500	––	–	–	–	–	–	2670	27 213
**Mn**	620	80	1200	–	–	–	–	–	–	2910	1808
**Mo**	–	–	–	–	–	–	–	–	–	–	245
**Ni**	46 360	–	30	6800	950	3300	–	–	–	–	99
**Pb**	55 840	20 030	–	9900	2300	16 000	5311	11 482	19 354	350	1744
**Sb**	–	–	–	–	–	–	–	–	–	–	241
**V**	–	–	70	–	–	–	–	–	–	–	71
Site type	industrial	urban industrial	coastal	urban	rural	coastal watersheds	rural	suburban	urban	mountain	coastal
Time period	2002–2003	2009–2010	2008–2011	2003–2005	–	–	–			2017–2021	Apr-Dec 2019
Location	Bursa Turkey	Taiwan	Capo Cuittone Corsica	Tokyo Bay Japan	New Zealand	Lihe River region China	Shanghai China			Mt Emei China	Gdynia Poland

In this study, the annual dry deposition flux of trace elements in the coarse particle fraction is defined as the total mass in µg deposited per m^2^ during the year, following the order: Fe > Mn > Pb > Cr > Cu > As > Mo ≥ Sb > Ni > V > Cd ≥ Co. These deposition values are substantially lower than those reported by Chen et al.^17^ in China, Gray et al.^31^ in New Zealand, and Sakata et al.^32^ in Japan, as shown in Table 1. The relatively high atmospheric inputs of As and Pb were observed in Shanghai, China, at urban, suburban, and rural locations^15^. The results of annual deposition fluxes for As, Cd, and Cu in Gdynia align with the deposition range reported in Fu et al.^*18*^ for the mountain region in China. Additionally, the vanadium deposition value at our coastal urban site is in better agreement with observations at the remote coastal site in Cap Cuittone, Corsica^16^, where V in the atmosphere primarily originates from mineral dust, biomass burning, and fossil fuel combustion. However, the deposition values of Cd, Co, Cr, Cu, Ni, and Pb in Gdynia were significantly lower than those in the industrial area of Bursa, Turkey^33^. In contrast, Gdynia’s annual manganese deposition flux was almost 3 and 23 times higher than observations in Turkey^33^ and Taiwan^34^, respectively. Our study revealed that atmospheric deposition fluxes of trace elements are significantly lower than those above urban and industrial regions in China, Turkey, and Japan, implying that the global atmospheric budget of trace elements is variable and depends on emission sources and meteorological conditions.

### Seasonal variation

**Fig. 2:**
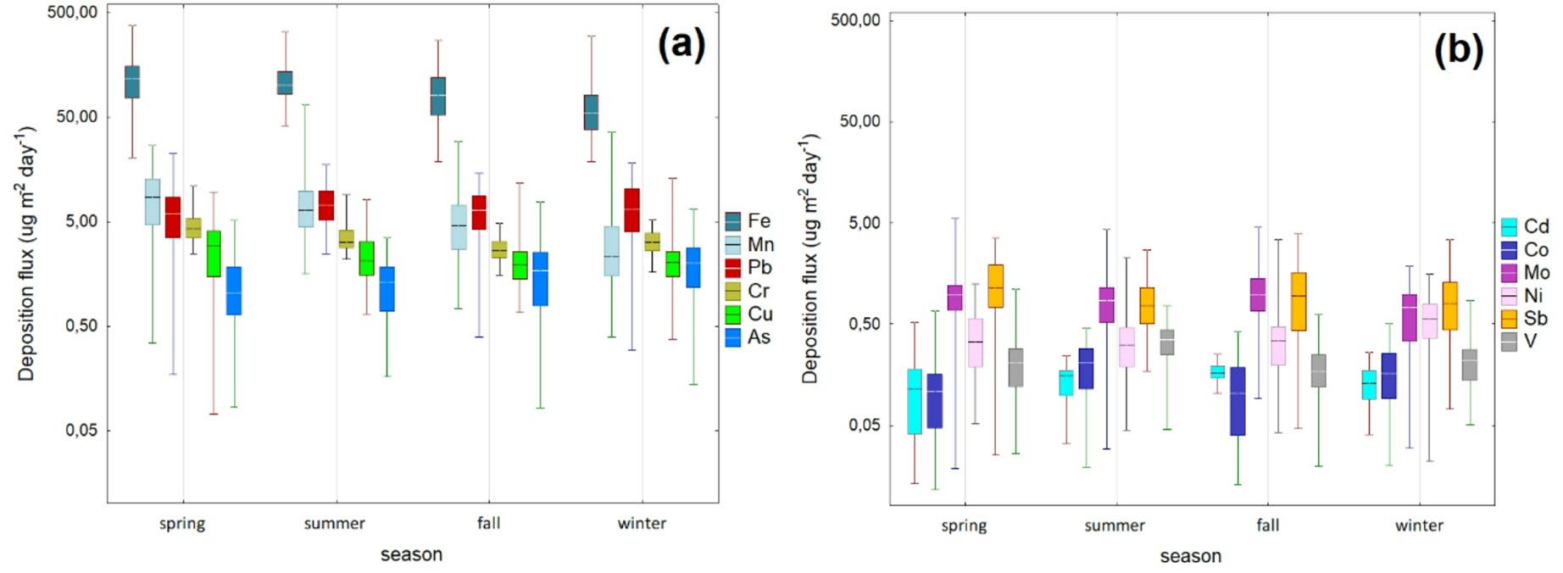
Box plots for the seasonal profiles of median dry deposition fluxes of (**a**) Fe, Mn, Pb, Cr, Cu, As, and (**b**) Cd, Co, Mo, Ni, Sb, V with coarse-mode particles PM_10_. The median dry deposition fluxes are displayed as a line inside boxes. The whiskers extend to the minimum and maximum values, while the boxes denote the 75th percentile (upper) and 25th percentile (lower). Statistically significant differences were found between seasons for 11 deposition fluxes (except Pb) (Kruskal-Wallis test, *p* < 0.05). Note that the Y-axis is shown in a logarithmic scale to facilitate the visualization of a wide range of deposition fluxes.

Figure 2 presents seasonal variations of dry deposition fluxes of trace elements as box-whisker plots. The daily median deposition of Cd in coarse particle fraction in spring (0.12 µg m^− 2^ day^− 1^) was slightly lower than that in other sampling seasons (summer mean: 0.15 µg m^− 2^ day^− 1^, fall mean: 0.16 µg m^− 2^ day^− 1^, winter mean: 0.13 µg m^− 2^ day^− 1^). The daily median deposition flux of Co (0.11 µg m^− 2^ day^− 1^) in spring was also significantly lower (*p*-value < 0.05) compared to summer (0.21 µg m^− 2^ day^− 1^) and winter (0.16 µg m^− 2^ day^− 1^), but not in fall (0.10 µg m^− 2^ day^− 1^). The hierarchical cluster analysis revealed that Co and Cd have the two most similar deposition profiles (Fig. S2 in the Appendix). The Fe seasonal deposition profile exhibits a steady decrease trend in this study (Fig. 1a). The median deposition flux of Fe in spring was higher (115.47 µg m^− 2^ day^− 1^, *p*-value < 0.05) than in summer (100.50 µg m^− 2^ day^− 1^), fall (80.78 µg m^− 2^ day^− 1^), and winter (54.94 µg m^− 2^ day^− 1^). Beyond that, it is substantially lower than the value reported by Zhang et al.^34^ in central Taiwan (Fe mean dry deposition flux: 400 µg m^− 2^ day^− 1^). Fe is an anthropogenic and crustal origin element that may be attributed to greater soil and road dust emissions in spring in Gdynia.

The median dry deposition flux of Cr decreased in the following order: 4.29 µg m^− 2^ day^− 1^ (spring) > 3.19 µg m^− 2^ day^− 1^ (summer) > 3.16 µg m^− 2^ day^− 1^ (winter) > 2.61 µg m^− 2^ day^− 1^ (fall). The median Cu dry deposition flux was significantly higher in spring (2.91 µg m^− 2^ day^− 1^) than in other seasons (summer median: 2.10 µg m^− 2^ day^− 1^, winter median: 2.04 µg m^− 2^ day^− 1^, fall median: 1.94 µg m^− 2^ day^− 1^). According to other studies in the coastal Mediterranean region^16^, the high deposition of Cu in the coarse mode may reflect sources such as biomass burning, waste disposal, and non-ferrous metal smelting. Another point to note is that the median Sb deposition flux in spring (1.14 µg m^− 2^ day^− 1^) was also significantly higher than in fall (0.95 µg m^− 2^ day^− 1^), winter (0.80 µg m^− 2^ day^− 1^), and summer (0.75 µg m^− 2^ day^− 1^). The median dry deposition flux of V was substantially higher in summer (0.35 µg m^− 2^ day^− 1^), followed by winter (0.22 µg m^− 2^ day^− 1^), spring (0.21 µg m^− 2^ day^− 1^), and fall (0.17 µg m^− 2^ day^− 1^). The median dry deposition flux of Pb was the highest in summer (7.16 µg m^− 2^ day^− 1^), followed by winter (6.48 µg m^− 2^ day^− 1^), fall (6.41 µg m^− 2^ day^− 1^), and spring (5.87 µg m^− 2^ day^− 1^). As for arsenic (As), its deposition flux increased significantly in winter (median: 2.00 µg m^− 2^ day^− 1^). Recently, based on positive matrix factorization analysis, Li et al.^35^ found that As in particles can be substantially enhanced by coal combustion for domestic heating during wintertime. The seasonal deposition profile of arsenic in our study shows similar characteristics. Figure 2a exhibits Mn’s highest dry deposition flux in spring (median: 8.48 µg m^− 2^ day^− 1^), almost 4 times higher than in winter. In the present study, the median atmospheric fluxes of Mo varied between 0.74 µg m^− 2^ day^− 1^ (winter) and 0.98 µg m^− 2^ day^− 1^ (spring), while for Ni, it was between 0.31 µg m^− 2^ day^− 1^ (summer) and 0.56 µg m^− 2^ day^− 1^ (winter). A similar seasonal pattern for Ni atmospheric deposition has been observed in the Lihe River watershed, located in the coastal region of eastern China^17^.

**Fig. 3:**
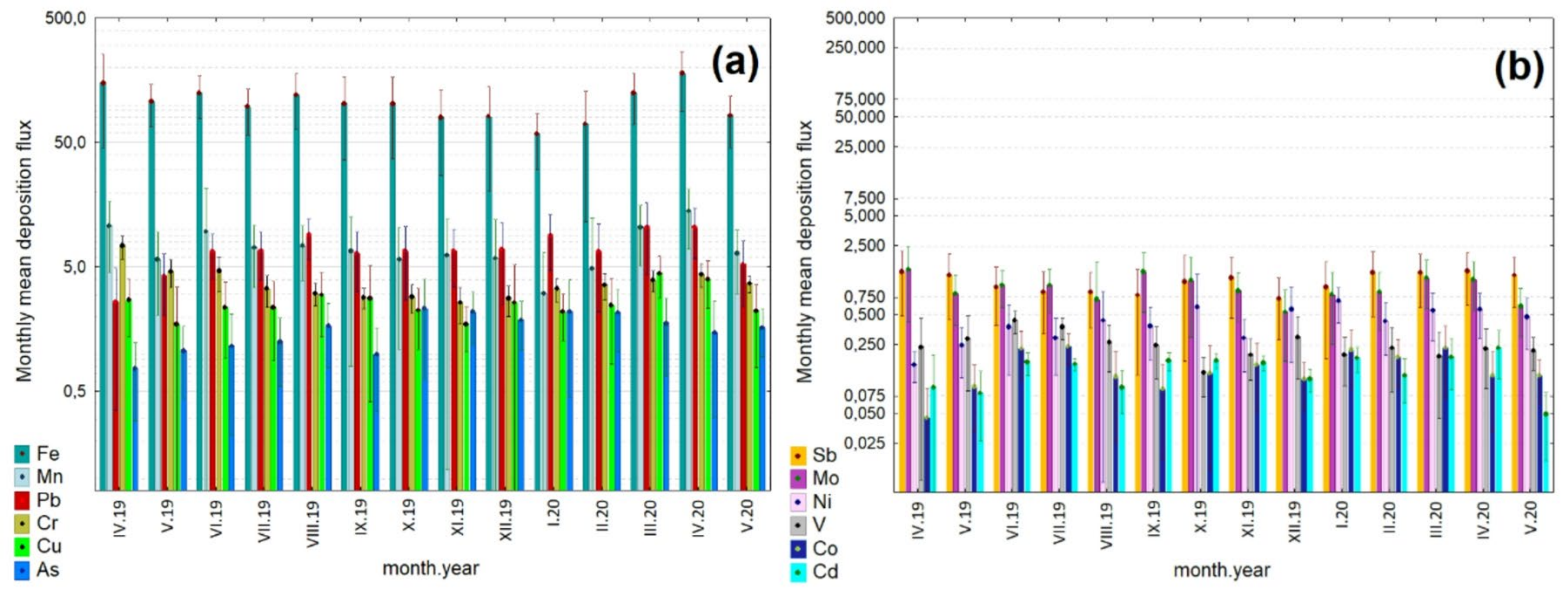
Monthly-scale temporal variability in mean deposition fluxes of (**a**) Fe, Mn, Pb, Cr, Cu, As, and (**b**) Sb, Mo, Ni, V, Co, Cd in the coarse particle fraction in Gdynia. The whiskers show ± 1 standard deviation of the mean. The y-axis is different across the figures. Note that the Y-axis is shown in a logarithmic scale to facilitate the visualization of a wide range of deposition fluxes.

The monthly changes were examined to accurately describe the variability of atmospheric deposition of anthropogenic trace elements during the study period (Fig. 3). The most abundant component in deposition flux for all months was Fe. As shown in Fig. 3a, the monthly mean dry deposition flux of Fe peaked at 179.27 ± 89.52 µg m^− 2^ in April 2020, three times higher than its value observed in January 2020. Previous studies^36^ identified the significant influence of non-exhaust traffic-related emissions on Fe mass concentration. Bukowiecki et al.^37^ displayed high values of Fe in the PM_10_ fraction associated with the traffic-related dust source. Furthermore, some elements (i.e., Fe, Cr, Cu, and Mn) were revealed as the most abundant trace elements in brake pads and brake lining, and thus can be attributed to traffic-related emissions^38^. There was a significant decreasing trend between April and November for Cr deposition (Fig. 3a), and between April and September for Sb deposition (Fig. 3b). A relatively high concentration of Sb-containing compounds in urban PM_10_ can be predominantly associated with abrasion products emitted by vehicles^39,40^. Our results align with previous research, suggesting that road traffic is a significant primary source of Sb in urban areas. For instance, Rossini et al.^41^ showed Sb deposition flux of 0.235 µg m^− 2^ d^− 1^ at the urban background site in Venice (Italy), while Langner et al.^42^ found a mean Sb deposit ranging between 34 and 124 µg m^− 2^ d^− 1^ at the roadside site in Karlsruhe (Germany) characterized with a traffic flow of about 100,000 vehicles per day. During the cold study period (October to March), the monthly mean value of the deposition flux of As ranged from 2.31 ± 1.68 to 1.78 ± 1.08 µg m^− 2^ (Fig. 3a). Ni’s monthly mean deposition flux exhibited the highest value of 0.68 ± 0.27 µg m^− 2^ in January 2020, followed by October 2019 (mean: 0.60 ± 0.71 µg m^− 2^), while the lowest mean Ni deposition flux of 0.16 ± 0.05 µg m^− 2^ was observed in April 2019 (Fig. 3b). Such a pattern for Ni deposition flux can be attributed to the proximity of commercial harbors and refinery plants. Ni may be associated with the combustion of heavy oil (i.e., in oil refineries, municipal wastewater sludge incinerators, thermal-electrical plants, the petrochemical industry, and large ship engines), as well as domestic heating. As shown in Fig. 3b, the monthly peak for Mo of 1.44 ± 1.02 µg m^− 2^ was found in April 2019, and the minimum monthly flux was determined in December 2019 (mean: 0.53 ± 0.36 µg m^− 2^). The highest monthly mean of Cd, Co, and Cu were 0.23 ± 0.12 µg m^− 2^ (April 2020), 0.24 ± 0.08 µg m^− 2^ (July 2019), and 4.47 ± 1.65 µg m^− 2^ (March 2020), respectively. The monthly deposition flux of Mn ranged from 3.07 ± 3.47 µg m^− 2^ (January 2020) to 14.13 ± 7.20 µg m^− 2^ (April 2020), with an average of 8.10 ± 5.33 µg m^− 2^ (Fig. 2a). Manganese is a good marker of road traffic emissions, while industrial emissions are marked by lead^5^. In the present study, the mean Pb deposition flux presented the highest level in March 2020 (10.41 ± 6.04 µg m^− 2^). A level almost the same as that observed in April 2020 (10.36 ± 4.54 µg m^− 2^) was also noted, which could imply that local Pb sources, including industrial processes, domestic heating activities, municipal solid waste incineration, and vehicle exhaust, were similarly significant contributors to Pb in PM_10_. A recent study by He and co-authors^43^ indicated that coal combustion was the primary contributor to Cr, Mn, and As in urban deposition in Lanzhou City (northwestern China); Pb was the dominant component in PM deposition from vehicles; As and Cd mainly originated from waste incineration, while high Cr and Cd fluxes were attributed to biomass burning.

Since the present study covers the spring 2020 COVID-19 lockdown period, differences in the deposition profiles of selected elements were also examined. As shown in Fig. 3a, the monthly deposition fluxes of Fe, Mn, Cu, and Pb exhibited a significant increase during the COVID-19 pandemic. In addition, a minor increase was observed for Mo, Cd, Ni, and Co (Fig. 2b), whereas the deposition profile of Cr, Sb, and V was largely consistent. The opposite trend was reported only for As deposition (Fig. 3a), which can be attributed to agricultural activities (i.e., the use of sulfate fertilizers and inorganic pesticides)^44^ and industrial activities. The effect of lockdown restrictions on trace element dry deposition fluxes in the coastal region in northern Poland did not show any notable variation, as expected. The results indicated that during the COVID-19 lockdown period in the coastal area of the Baltic Sea (February-April 2020), meteorological conditions were favorable for pollutant dispersion (Table S1). As mentioned above, the selected elements are related to various anthropogenic sources, including road traffic, industrial emissions, waste incineration, domestic heating, shipping activities, and petrochemical refineries. However, the reduction in emission rates for these sectors and their relationships with PM remain complex and varied, but also dependent on local meteorology, atmospheric processes, and site characteristics. Recent work by Putaud et al.^45^, focusing on the impact of lockdown measures on particulate air pollution at urban and regional background sites across Europe, also highlighted the complexity of PM mass concentration responses to the COVID-19 lockdown measures, which arise from a combination of several factors.

### Meteorological drivers

 The influence of meteorological parameters, including ambient air temperature, air pressure, wind velocity, and relative humidity, on trace element dry deposition fluxes was examined based on Spearman’s *R* correlation coefficient (Table 2). Specifically, PM_10_ did not correlate notably with air pressure or relative humidity in Gdynia. Similar results were observed between the deposition fluxes of Cd, Mo, Pb, and Sb and the selected meteorological variables. As shown in Table 2, the impact of air pressure was statistically significant but minimal for Co (*R* = -0.202), Mn (*R* = 0.146), Fe (*R* = 0.136), As (*R* = -0.132), Cu (*R* = 0.115), and Cr (*R* = 0.109). Coastal PM_10_ was significantly correlated with Fe (*R* = 0.471), Mn (*R* = 0.353), and a slightly lower positive correlation was found between coarse particles and Cu (*R* = 0.283), Pb (*R* = 0.229), and Cr (*R* = 0.190). Some trace elements, e.g., V (*R* = 0.364), Fe (*R* = 0.280), and Mn (*R* = 0.226), showed weak positive correlations with temperature (*p*-value < 0.05; Table 2). Interestingly, temperature was negatively correlated at a significance level of *p*-value < 0.05 with As (*R* = -0.177) and Ni (*R* = -0.181), indicating an impact of thermal inversion as reported by Bodor et al.^46^. The relatively high correlation was found between Fe deposition and Mn deposition (*R* = 0.830, *p* < 0.05), between Mn deposition and Cu deposition (*R* = 0.618, *p*-value < 0.05), and between Fe deposition and Cu deposition (*R* = 0.624, *p* < 0.05). A possible explanation for the positive correlations between Fe and Mn is the occurrence of at least one dominant source of these metals, which could be due to dust resuspension and road surface abrasion. For instance, Visser et al.^47^ found that 65% of the coarse fraction Fe can typically be attributed to traffic-related mechanical abrasion processes, while coarse fraction Mn and Cr are also good indicators of industrial processes. The statistically significant correlation was found between relative humidity and atmospheric deposition of some trace elements: Fe (*R* = -0.354), Cr (*R* = -0.302), Mn (*R* = -0.289), As (*R* = 0.192), Cu (*R* = -0.184), Ni (*R* = 0.176), and V (*R* = -0.124), suggesting a role of air moisture and aqueous-phase aerosol chemistry in the transfer of these compounds from the atmosphere to the coastal region. The matrix correlation analysis revealed statistically significant and negative Spearman *R* values when evaluating the relationships between wind speed and the deposition of Cr, Cu, Fe, Mn, and V. Thus, wind velocity was identified as a significant environmental factor affecting the deposition processes of these elements. The positive and significant correlation between deposition of Pb-Cu (*R* = 0.446), Pb-Mn (*R* = 0.280), Pb-Cd (*R* = 0.273), and Pb-Fe (*R* = 0.302) may suggest mixed source origins, including industrial processes, traffic-related and resuspended dust, fossil fuel/coal combustion, and biomass burning. Studies in China^17^ showed that Cr in atmospheric deposition was highly correlated with Pb (*R* = 0.911), similar to Pb and Cd (*R* = 0.681) at the *p* = 0.05 level. In contrast, other metals were weakly correlated^17^. The correlation results in this study correspond only to some findings from the mentioned study, reflecting that the contribution of mixed primary sources, including their strength, location, and meteorological conditions, to the chemical composition of PM_10_ and its depositional processes is significant and pronounces large variability. The multiple regression analysis, considering all meteorological parameters as predictor variables, identified relative humidity and wind speed as the most significant predictors of Fe deposition (*p* = 0.000, Table S4). The MLR explained 14.1% of the variability in Fe deposition (R^2^ = 0.141), suggesting a weak meteorological impact on deposition. The multiple regression model also showed that V deposition was significantly influenced by air temperature (*p* = 0.000, Table S4). These findings suggest that the influence of meteorology on deposition processes is relatively complex and may depend on other parameters, such as meteorological anomalies, changes in atmospheric circulation, seasonal coastal processes, emission sources, and pollution rates.

**Table 2:** The spearman correlation matrix of element deposition fluxes (data log-transformed) and meteorological variables. Correlations statistically significant at 95% are presented in bold. The symbols are as follows: p – air pressure, T – ambient temperature, RH – relative humidity, WS – wind speed.

Variable	PM_10_	As	Cd	Co	Cr	Cu	Fe	Mn	Mo	Ni	Pb	Sb	V	*p*	T	RH
As	−0.029															
Cd	0.046	0.058														
Co	−0.044	0.128	**0.305**													
Cr	**0.190**	**−0.195**	−0.018	−0.016												
Cu	**0.283**	0.013	**0.182**	0.040	**0.301**											
Fe	**0.471**	−0.078	**0.111**	0.003	**0.448**	**0.624**										
Mn	**0.353**	−0.103	**0.109**	0.003	**0.460**	**0.618**	**0.830**									
Mo	0.046	−0.118	**0.313**	0.066	**0.153**	0.082	0.091	0.058								
Ni	−0.071	**0.210**	**0.141**	0.086	−0.017	**0.201**	0.010	0.010	−0.050							
Pb	**0.229**	0.021	**0.273**	**0.165**	0.070	**0.446**	**0.302**	**0.280**	−0.051	**0.253**						
Sb	0.096	0.044	0.045	−0.004	0.079	0.112	0.098	0.016	0.054	−0.114	−0.005					
V	**0.151**	**−0.122**	**0.121**	**0.157**	**0.171**	**0.150**	**0.283**	**0.233**	0.034	0.010	**0.114**	−0.020				
p	0.099	**−0.132**	0.080	**−0.202**	**0.109**	**0.115**	**0.136**	**0.146**	0.070	−0.030	0.052	−0.069	−0.043			
T	**0.221**	**−0.177**	0.010	0.092	0.054	−0.049	**0.280**	**0.226**	0.068	**−0.181**	−0.022	−0.087	**0.364**	−0.018		
RH	−0.060	**0.192**	0.104	0.130	**−0.302**	**−0.184**	**−0.354**	**−0.289**	−0.088	**0.176**	0.094	−0.110	**−0.124**	**−0.330**	**−0.360**	
WS	**−0.210**	−0.012	−0.014	−0.017	**−0.110**	**−0.141**	**−0.255**	**−0.193**	−0.001	0.025	−0.081	−0.048	**−0.133**	−0.078	**−0.381**	0.093

### Importance of wind clusters

**Fig. 4:**
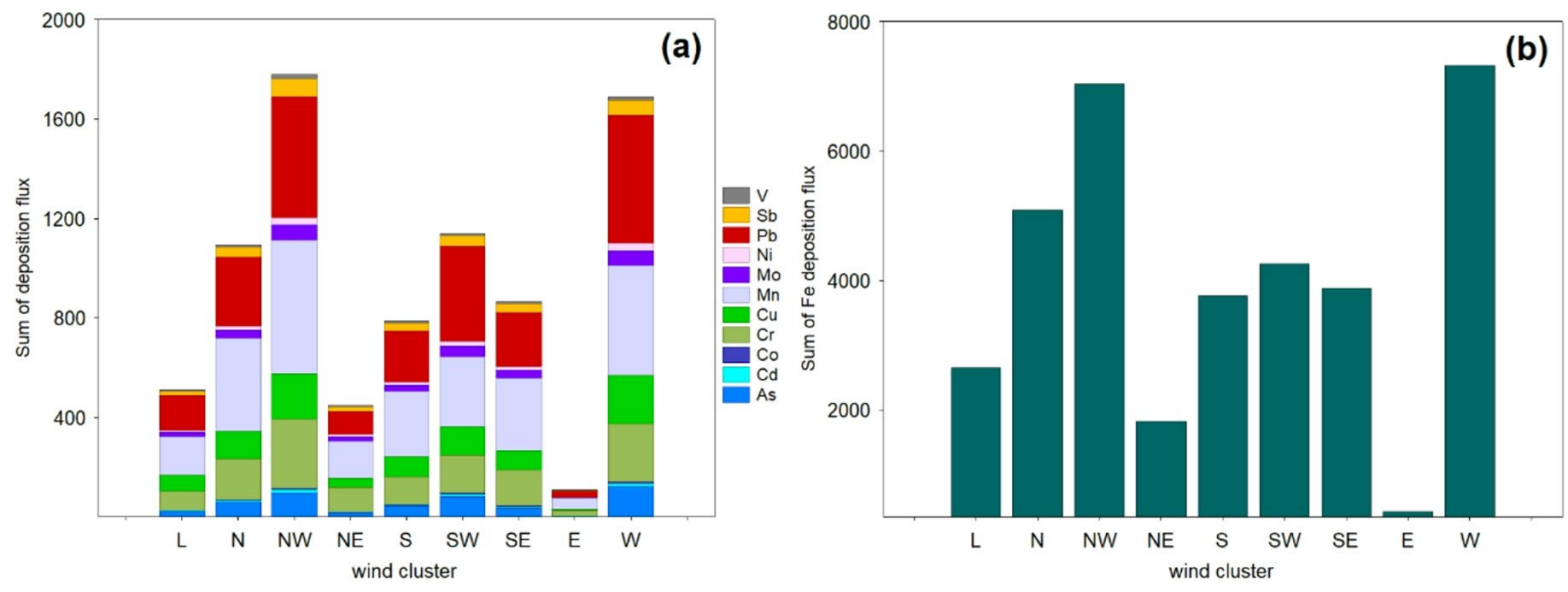
Total sum of deposition fluxes of (**a)** 11 trace elements (F_11_) and (**b)** Fe in PM_10_ contributed by wind clusters. Note that the y-axis is different across the figures. For each deposition flux, a Kruskal–Wallis one-way analysis of variance (ANOVA) on ranks among clusters was performed at *p* < 0.05. Significant differences were found in all clusters for the following trace element depositional fluxes: As (Kruskal–Wallis test, *p* = 0.018), Cr (Kruskal-Wallis test, *p* = 0.0001), Fe (Kruskal-Wallis test, *p* = 0.0079), Mn (Kruskal-Wallis test, *p* = 0.0015), Ni (Kruskal-Wallis test, *p* = 0.0091), and V (Kruskal-Wallis test, *p* = 0.0000).

 When analyzing the contribution of air masses transport pathways to the sum of deposition fluxes of trace elements, a pronounced variability was found between the observed deposition fluxes of elements related to W-NW and other wind clusters (Fig. 4). As presented in Table S2, clusters W and NW contributed to 21% of the atmospheric circulation, indicating dominant sources that shape atmospheric TE concentrations and deposits in the coastal region of the northern Poland.

Remarkably, the total deposition flux of 12 components in coarse coastal particles associated with the N air flow transport plume was higher than in the NE cluster. As shown in Fig. 4a, under NE regime, representing transport of air masses originating from the Gulf of Gdansk, the target study region experienced relatively low atmospheric deposition fluxes of 11 trace elements (F_11_ = 448.22 µg m^− 2^), while under a more polluted regimes (NW and W clusters), the high deposition fluxes driven by inland industrial and commercial coal combustion emissions were observed (F_11_ = 1779.35 and 1690.76 µg m^− 2^, respectively). The same trend was observed concerning Fe deposition fluxes (Fig. 4b). Deposition fluxes of transition metals, such as Cu, were slightly higher in the S cluster than in the SE cluster, suggesting that the impact of the Cu emission from major anthropogenic hotspots (i.e., coal combustion, industrial activities) is much higher than that of local vehicles. Under the SE-S-SW regimes, the total deposition fluxes of 11 elements (without Fe) ranged from 789.81 µg to 1141.61 µg m^− 2^. Figure 4a shows that during the north long-range transport plumes, the deposition fluxes of As, Cr, Mn, Ni, Sb, and Pb exhibit significantly higher values compared with S and SE clusters, suggesting the polluting effect of the PM_10_ at our coastal site could reflect the notable variation of local and regional emissions. However, wind speed is a crucial parameter that shows which contribution (local or regional) is predominant. In this study, the majority of high PM_10_-bound trace element deposition fluxes occurred during relatively low and stable wind conditions, suggesting a significant contribution from local sources. However, influences from distant NW and W Europe regions were also substantial. In summary, the transport cluster’s impact on trace elements’ deposition fluxes is significantly diverse and affected by essential features of the air mass advection regime, regional sources, local anthropogenic activity, wind speed and direction.

### Source identification

 The source apportionment based on principal component analysis (PCA) was applied to the normalized values of the daily deposition fluxes of 12 elements and PM_10_. This analysis reveals that four principal components (PC_1 − 4_) account for 55% of the variability within the measured dataset (Table 3). Only eigenvalues > 1 were selected for PCA (Fig. S3). Specifically, the first principal component (22% of the total variance) exhibits high factor loadings for Fe (0.784), followed by Mn (0.717) and Cu (0.682), indicating that PC_1_ represents mixed sources controlling a high level of these compounds in the coastal atmosphere, including road- and soil-related dust re-suspension (Mn, Cu, Fe). Previous studies by Li et al.^48^ have shown that transportation sources may contain a high loading of Mn, which is regarded as a tracer of brake wear. For PC_2_ (13% of the total variance, Table 3), the negative high loading of As deposition (-0.787) and positive high loading of V deposition (0.750) suggest a significant contribution of coal-fired power plants. PC_3_ (11% of the total variance) exhibits a high positive loading of Cd (0.745) and Co (0.749), suggesting high-temperature commercial oil combustion in refineries during local events. The fourth component (PC_4_), representing 9% of the total variation, is associated with Sb (*R* = 0.589) and Mo (*R* = 0.581), suggesting that road traffic may be the primary source of these elements. In previous studies in Sweden^49^, these elements have been identified as significant indicators of road wear, as well as in industries such as incineration, wastewater treatment, and biomass combustion. Similar results from PCA analysis provide evidence that contributions from different local source emissions significantly affect particle fluxes in the coastal region of the Gulf of Gdansk.

**Table 3:** Principal component analysis (PCA) with varimax rotation for the input deposition variable of PM_10_ and 12 elements at the coastal site in Gdynia, Poland. Data were standardized.

Variable	PC_1_	PC_2_	PC_3_	PC_4_
PM_10_	0.568	0.102	−0.086	0.310
As	0.084	**−0.787**	0.000	0.088
Cd	0.112	0.002	**0.745**	0.023
Co	−0.151	0.075	**0.749**	0.003
Cr	0.514	0.506	0.108	0.227
Cu	**0.682**	−0.210	0.120	−0.193
Fe	**0.784**	0.243	−0.089	0.203
Mn	**0.717**	0.104	−0.075	−0.065
Mo	0.131	0.008	0.360	0.581
Ni	−0.029	−0.278	0.185	−0.476
Pb	0.500	0.008	0.381	−0.467
Sb	0.347	−0.154	0.048	0.589
V	0.205	**0.750**	0.073	0.092
*eigenvalue*	2.7	1.6	1.5	1.2
*% of variance explained*	22%	13%	11%	9%

**Fig. 5:**
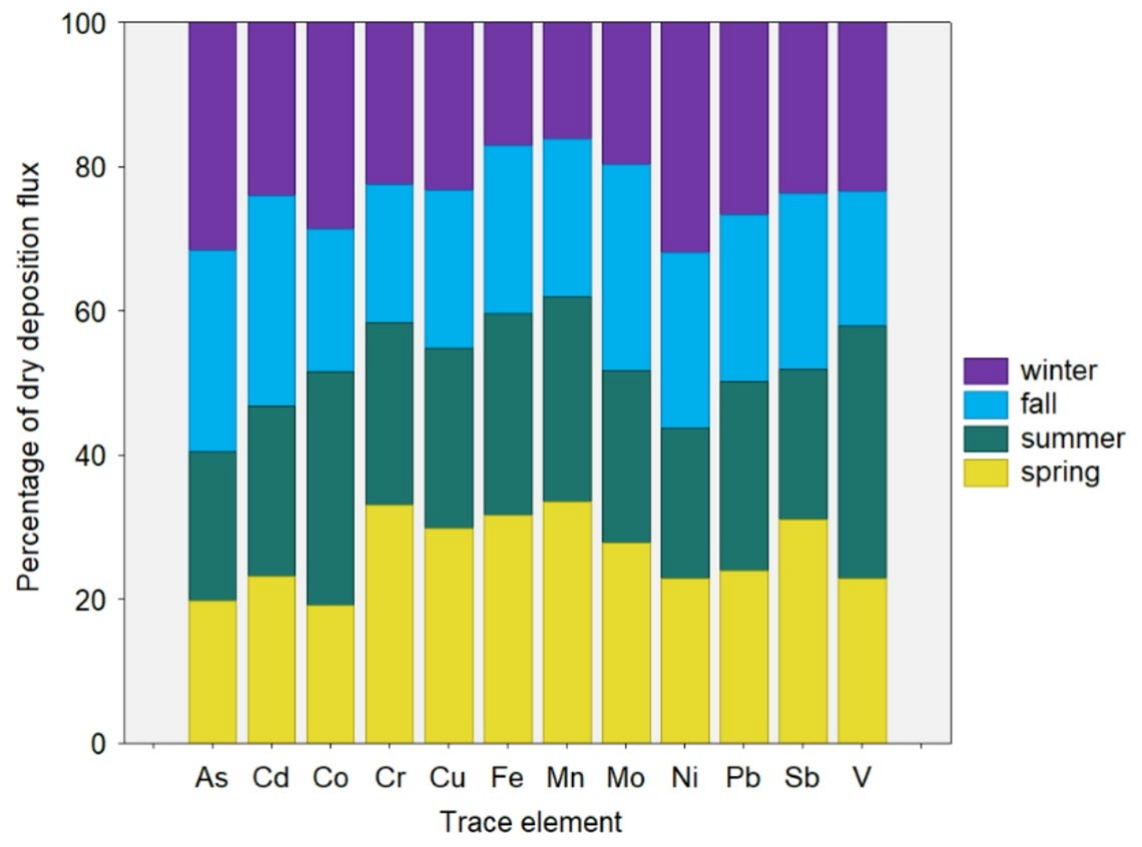
Relative distribution of dry deposition fluxes of individual trace elements contributed by four seasons.

A distribution of deposition fluxes exhibits different patterns depending on meteorology and emission sources^2^. Figure 5 shows the seasonal variation of As, Cd, Co, Cr, Cu, Fe, Mn, Mo, Ni, Pb, Sb, and V fluxes from PM_10_ aerosols. A clear interannual variability, as a combination of source emissions and meteorology, generally characterizes the distribution of trace element fluxes during the sampling period in this region. Overall, the Ni and As contributions at the coastal site in Poland were typically lower in spring but higher in winter, indicating the impact of specific emission sources (i.e., industrial combustion, coal combustion for household heating). It should also be noted that during the winter period (DJF), there are more favorable meteorological conditions (Table S1), including low boundary layer height, low air temperature, and high relative humidity, the occurrence of the thermal inversion layer compared with summer period (JJA), which, combined with intensive local and regional heating activities, may significantly enhance high concentrations of PM_10_-bound trace elements over the study domain. The same seasonal behavior for Ni and As was observed by Waked et al.^50^ in Lens, northern France. To investigate the impact of different anthropogenic sources on the molecular composition of PM_10_, the mean values of the diagnostic concentration ratios for several pairs of metals were considered: Cu/Sb, V/Ni, Cu/Pb, Cd/Cu, and Mn/V. Since various primary sources influence the study domain, it is likely that the impact of industrial high-temperature combustion emissions, motor vehicle emissions, and abrasion from tires or brake linings, as well as the activities of refineries, shipping, docks, and ports located in this area, is significant. In particular, the Cu/Sb ratio (ranging from 1.13 to 8.33) is a good indicator for brake wear-related emissions. The Cu/Sb ratio of 5.79 in this study was similar to the values reported by Sternbeck et al.^51^ in Switzerland (5.35 ± 2.9), indicating a significant contribution from traffic emissions. The contribution of V at 35% was predominantly observed in summer, most probably due to emissions from the coastal port and marine shipping (Fig. 5). These findings are consistent with studies, such as those by Ealo et al.^52^, Wang et al.^53^, and Chatoutsidou et al.^54^, showing that shipping and coastal activities (i.e., petrochemical refining) significantly impact total V and Ni emissions in the port area. Vanadium and nickel are two of the main constituents of particulate matter associated with heavy oil combustion. Previous studies reported that the V/Ni ratio of 3–4 (Mazzei et al.^55^) predicts heavy oil combustion in the petroleum refinery industry as one primary emission source of these metals; their lower values indicate oil combustion (Peltier et al.^56^), while ratios of 2.5-5 suggest ship emissions (Salameh et al.^57^). A study by Becagli et al.^58^ also showed a large content of V and Ni in marine plume transport. Other studies^59^ showed that the high contribution from industrial and shipping emissions can be identified by diagnostic concentration ratios of V/Ni, which range from 2.3 to 4.5. The value of V/Ni (1.68) was slightly lower in this study, indicating mixed sources in Gdynia. The diagnostic ratio of Cu/Pb in this study, at 1.19, fell within the typical range of traffic emissions. In contrast, the ratio of 0.23 for Cd/Cu may indicate the influence of waste incineration, as mentioned by Font et al.^60^. The ratio of Mn/V of 5.35 in Gdynia is in agreement with values reported at the coastal city in Colombia^61^, suggesting a significant influence from coal burning.

As shown in Fig. 5, the main elements in deposition fluxes in winter differ significantly from those in summer. In winter, local influences, primarily from commercial (e.g., coal power plants) and domestic sources, reach their highest levels in this region^21,62^. Data analysis reveals that for Ni and As, their contribution increased substantially to more than 30% in winter, followed by Co (29%), Pb (27%), Cd, and Sb (24%), which is due to stronger emission of these compounds during the cold study period, comparable to results reported in previous studies in this region. A relatively high concentration of Sb-containing compounds in urban PM_10_ can be predominantly associated with abrasion products emitted by vehicles^39,40^. A similar seasonal trend of dry deposition flux was observed for Cr (spring: 33% > summer: 25% > winter: 22% > fall: 19%), Cu (spring: 30% > summer: 25% > winter: 23% > fall: 22%), Fe (spring: 32% > summer: 28% > fall: 23% > winter: 17%), and Mn (spring: 34% > summer: 28% > fall: 22% > winter: 16%). The seasonal distribution analysis shows that spring maxima of Fe and Mn were related to dust resuspension and road surface abrasion. As shown in Fig. 5, Mo’s highest and lowest seasonal mean contributions to the annual deposition flux were observed in fall (29%) and winter (19%), respectively. These results suggest that the distribution and magnitude of dry deposition fluxes of elements are attributed to changes in the dominant anthropogenic sources, the chemical composition of PM_10_, and the seasons.

## Conclusions and implications

This study provides the first comprehensive characterization of the intra-seasonal variation of dry deposition fluxes based on daily PM_10_ field measurements conducted in the coastal region of northern Poland, focusing on 12 elements: As, Cd, Co, Cr, Cu, Fe, Mn, Mo, Ni, Pb, Sb, and V. The HYSPLIT model and principal component analysis (PCA) were leveraged to estimate air mass transport and identify profiles and sources of the aforementioned compounds in PM_10_. Four primary urban sources were inferred from PCA, including the first group, represented by road- and soil-related dust re-suspension (22%), the second by coal-fired power plants (13%), the third by high-temperature commercial oil combustion in refineries (11%), and the fourth, with a significant role of traffic emissions (9%). The main finding from this study is that the increase in deposition fluxes of As was attributed to the S air flow regime, representing the local urban emissions. Deposition contribution from Cr (34%) was similar to that of Mn (33%), but greater than the contribution of other elements in spring. Lastly, the importance of Ni and As sources (i.e., industrial combustion and coal combustion for household heating) was notable, as identified in the winter results.

Due to the lack of direct field observations of deposition velocities for these elements within the study domain, all depositional fluxes were calculated based on constant values adopted from literature surveys, which may introduce some uncertainties. Although this study did not directly address these limitations, it still confirms the importance of deposition processes in evaluating the effectiveness of further air quality improvements in this region. Further research should also explore the significance of other meteorological factors, such as precipitation. Hence, additional measurements, combined with well-parameterized models, should reduce uncertainties in estimating trace element deposition. Such complex analysis might provide valuable feedback to the decision-making process in this region.

## Supplementary Information

Below is the link to the electronic supplementary material.

Supplementary Material 1

## Data Availability

All data generated and analyzed during this study are included in this article (and its supplementary files).

## Declarations

### Corresponding author

Correspondence to Patrycja Bukowska.
