# Supplementary material for: Seasonal characteristics and key sources of trace element deposition fluxes in coastal Poland
Source: Sci Rep. 2026 Jan 5;16:2317. doi: 10.1038/s41598-025-32170-z (PMC12816065; doi:10.1038/s41598-025-32170-z)

**Supplementary files**

**Seasonal characteristics and key sources of trace element deposition fluxes in coastal Poland**

Patrycja Bukowska^1^

E-mail: ^1^patrycja.bukowska@imgw.pl

Institute of Meteorology and Water Management, PL 80-342 Gdynia, Waszyngtona 42, Poland

**Figure S1** Location of the sampling point (SP) with surroundings in Gdynia, northern Poland. The dots represent major local anthropogenic sources: coal-fired power plants (red), port and dock areas (blue), petrochemical refineries and plants (green), municipal solid waste recycling units (yellow). The ESRI ArcGIS Desktop software, version 9.3, was used to process the data and create a map (on the left). The photos (on the right) show a sampling platform and set.


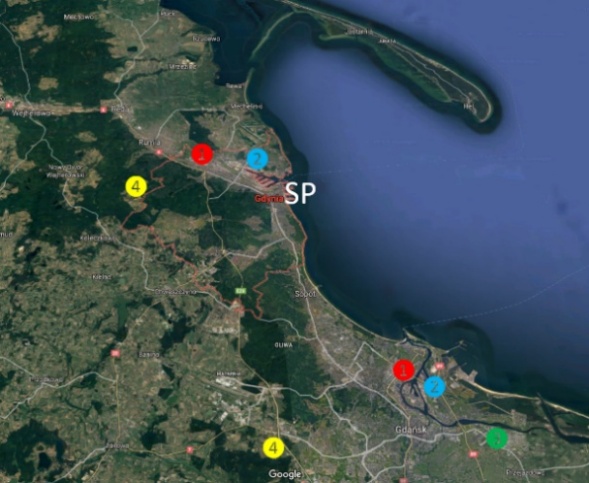

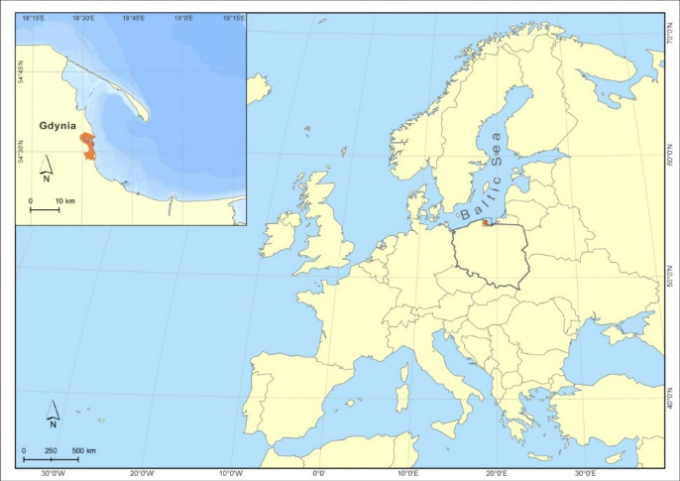


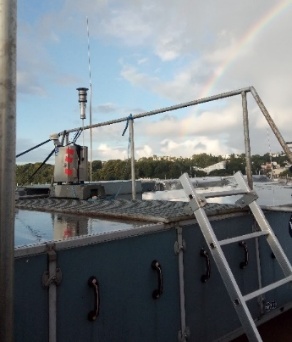

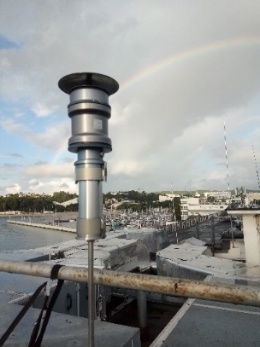


**Table S1.** Summary of meteorological data (mean air temperature, mean air pressure, mean relative humidity, and mean wind velocity) at the coastal sampling site (Gdynia, Poland) during the PM_10_ measurements.

| **Month/year** | **Period** | **Mean**  **air pressure**  **(hPa)** | **Mean**  **air temp**  **(°C)** | **Mean**  **relative**  **humidity**  **(%)** | **Mean**  **wind**  **velocity**  **(m s^-1^)** |
| --- | --- | --- | --- | --- | --- |
| 4/19 | AM | 1015.6 | 8.6 | 65.3 | 4.3 |
| 5/19 | AM | 1008.5 | 12.3 | 69.9 | 3.6 |
| 6/19 | JJA | 1012.8 | 20.8 | 69.0 | 3.5 |
| 7/19 | JJA | 1008.3 | 18.1 | 70.7 | 4.1 |
| 8/19 | JJA | 1011.6 | 20.5 | 67.9 | 3.2 |
| 9/19 | SON | 1011.7 | 14.0 | 75.7 | 4.2 |
| 10/19 | SON | 1008.5 | 10.2 | 81.9 | 3.5 |
| 11/19 | SON | 1005.1 | 5.3 | 89.5 | 5.2 |
| 12/19 | DJF | 1006.8 | 3.1 | 82.9 | 4.5 |
| 1/20 | DJF | 1012.5 | 3.9 | 83.5 | 4.2 |
| 2/20 | DJF | 1003.1 | 3.5 | 77.9 | 5.1 |
| 3/20 | MAM | 1013.8 | 4.3 | 72.8 | 4.3 |
| 4/20 | MAM | 1013.1 | 7.5 | 64.3 | 4.1 |
| 5/20 | MAM | 1009.3 | 8.6 | 71.2 | 3.9 |

**Table S2**. Contribution of air mass cluster to transport pathway over the study domain and characteristics of air mass source that arrive at the coastal city of Gdynia, northern Poland.

| Cluster | Contribution (%) | Characteristics |
| --- | --- | --- |
| **N** | 13 | This study defines the N cluster as the marine domain, where relatively clean air masses originating from northern European countries (i.e., Norway, Sweden, Finland) pass over the Baltic Sea area. |
| **NE** | 6 | The NE cluster represents the docks and ports area (shipping emission sector). |
| **E** | 1 | The E cluster contains the marine origin (the Gulf of Gdańsk coastal waters) and the approach fairway to the international port of Gdynia. |
| **SE** | 10 | The SE air flow trajectory cluster is associated with southeasterly air mass advection from the emission regions in Ukraine and Russia. |
| **L** | 5 | The local coal-fired power plant, located in the Gdańsk port and docks area, and a large complex of petrochemical refineries form part of the local cluster. |
| **S** | 9 | The S cluster is characterized by major industrial hotspots in Poland (Upper Silesia region) and the southern parts of Europe (i.e., the Balkans, Italy, Croatia, Romania, Hungary, the Czech Republic, and Slovenia), with noticeable influences from the Mediterranean Sea. |
| **SW** | 14 | The SW cluster includes local/regional traffic-related sources, residential heating, power generation, and industrial activities. Additionally, it highlights the impact of polluted areas in southwestern Europe, including the Czech Republic, Austria, Switzerland, and Spain. |
| **W** | 21 | The W cluster represents the local road network, residential areas, and municipal solid waste recycling units in central and western European countries (i.e., France, Germany, Austria), with various industrial/urban activities. |
| **NW** | 21 | The NW air flow cluster represents northwestern European areas (i.e., Ireland, the United Kingdom, Denmark, the Danish Straits, and northern Germany), as well as the North Sea, providing a mixture of maritime PM with trace gases and other pollutants. It also reflects the presence of a nearby coal-fired power plant and municipal solid waste incineration. |

**Table S3** Descriptive statistics for PM_10_ mass concentration and deposition fluxes (DFs) for each trace element obtained at the coastal urban site in Gdynia, Poland, during the sampling period April 2019 – May 2020. Data are given in μg m^-3^ (PM_10_) and μg m^-2^ day^-1^ (DFs).

|  | Mean | Standard deviation | Minimum | Maximum | 10%  percentile | 90%  percentile | Coefficient of variance (%) |
| --- | --- | --- | --- | --- | --- | --- | --- |
| PM | 17.7 | 10.49 | 3.40 | 65.52 | 6.90 | 32.76 | 59.3 |
| As | 1.61 | 1.13 | 0.08 | 7.75 | 0.40 | 2.95 | 70.2 |
| Cd | 0.14 | 0.08 | 0.01 | 0.52 | 0.04 | 0.22 | 52.5 |
| Co | 0.16 | 0.11 | 0.01 | 0.67 | 0.04 | 0.33 | 70.1 |
| Cr | 3.74 | 1.47 | 1.54 | 10.99 | 2.31 | 5.38 | 39.2 |
| Cu | 2.61 | 1.76 | 0.07 | 13.11 | 0.95 | 4.93 | 67.7 |
| Fe | 105.68 | 66.33 | 18.77 | 378.62 | 39.74 | 195.08 | 62.8 |
| Mn | 7.40 | 6.52 | 0.34 | 64.83 | 1.70 | 15.29 | 88.0 |
| Mo | 0.97 | 0.66 | 0.02 | 5.55 | 0.29 | 1.64 | 68.5 |
| Ni | 0.43 | 0.34 | 0.02 | 3.42 | 0.13 | 0.82 | 78.8 |
| Pb | 7.12 | 4.17 | 0.17 | 22.53 | 2.44 | 12.84 | 58.6 |
| Sb | 1.09 | 0.73 | 0.03 | 3.96 | 0.31 | 2.02 | 66.8 |
| V | 0.25 | 0.16 | 0.02 | 1.11 | 0.08 | 0.44 | 61.3 |

**Table S4** Multiple regression analysis results for individual dry deposition of elements and meteorological factors (i.e., ambient air temperature, air pressure, relative humidity, wind speed). Data marked in red represents meteorological factors statistically significant (*p*-value < 0.05) for deposition flux variation.

| Predictor variable | Coefficient b | Standard error | t-Statistic | *p*-value |
| --- | --- | --- | --- | --- |
|  | **PM_10_** | | | |
| Intercept | -104,259 | 64,856 | -1,608 | 0,109 |
| Air pressure | 0.122 | 0.062 | 1.964 | 0.050 |
| Air temperature | 0.185 | 0.098 | 1.899 | 0.058 |
| Relative humidity | 0.021 | 0.060 | 0.356 | 0.722 |
| Wind speed | -0.984 | 0.412 | -2.392 | 0.017 |
|  | **Arsenic (As)** | | | |
| Intercept | 10.905 | 7.339 | 1.486 | 0.138 |
| Air pressure | -0.009 | 0.007 | -1.329 | 0.185 |
| Air temperature | -0.029 | 0.011 | -2.583 | 0.010 |
| Relative humidity | 0.010 | 0.007 | 1.444 | 0.150 |
| Wind speed | -0.073 | 0.047 | -1.560 | 0.120 |
|  | **Cadmium (Cd)** | | | |
| Intercept | -0.824 | 0.473 | -1.744 | 0.082 |
| Air pressure | 0.001 | 0.000 | 2.045 | 0.042 |
| Air temperature | 0.000 | 0.001 | -0.428 | 0.669 |
| Relative humidity | 0.000 | 0.000 | 1.052 | 0.294 |
| Wind speed | 0.001 | 0.003 | 0.425 | 0.671 |
|  | **Cobalt (Co)** | | | |
| Intercept | 1.836 | 0.856 | 2.144 | 0.033 |
| Air pressure | -0.002 | 0.001 | -2.245 | 0.026 |
| Air temperature | 0.003 | 0.001 | 2.288 | 0.023 |
| Relative humidity | 0.002 | 0.001 | 2.052 | 0.041 |
| Wind speed | 0.001 | 0.006 | 0.193 | 0.847 |
|  | **Chromium (Cr)** | | | |
| Intercept | 7.019 | 8.797 | 0.798 | 0.426 |
| Air pressure | 0.001 | 0.008 | 0.100 | 0.920 |
| Air temperature | -0.025 | 0.013 | -1.879 | 0.061 |
| Relative humidity | -0.045 | 0.008 | -5.523 | 0.000 |
| Wind speed | -0.122 | 0.056 | -2.170 | 0.031 |
|  | **Cooper (Cu)** | | | |
| Intercept | -1.031 | 10.987 | -0.094 | 0.925 |
| Air pressure | 0.008 | 0.011 | 0.759 | 0.449 |
| Air temperature | -0.052 | 0.017 | -3.148 | 0.002 |
| Relative humidity | -0.040 | 0.010 | -3.898 | 0.000 |
| Wind speed | -0.210 | 0.071 | -2.980 | 0.003 |
|  | **Iron (Fe)** | | | |
| Intercept | -46.086 | 388.036 | -0.119 | 0.906 |
| Air pressure | 0.320 | 0.371 | 0.863 | 0.389 |
| Air temperature | -0.060 | 0.589 | -0.103 | 0.918 |
| Relative humidity | -1.748 | 0.359 | -4.870 | 0.000 |
| Wind speed | -9.591 | 2.489 | -3.854 | 0.000 |
|  | **Manganese (Mn)** | | | |
| Intercept | -34.759 | 40.391 | 0.861 | 0.390 |
| Air pressure | 0.051 | 0.039 | 1.333 | 0.184 |
| Air temperature | -0.014 | 0.061 | -0.226 | 0.821 |
| Relative humidity | -0.103 | 0.037 | -2.761 | 0.006 |
| Wind speed | -0.471 | 0.258 | -1.826 | 0.069 |
|  | **Molybdenum (Mo)** | | | |
| Intercept | -2.656 | 4.541 | -0.585 | 0.559 |
| Air pressure | 0.003 | 0.004 | 0.798 | 0.426 |
| Air temperature | 0.010 | 0.007 | 1.438 | 0.151 |
| Relative humidity | 0.000 | 0.004 | 0.073 | 0.941 |
| Wind speed | 0.000 | 0.028 | 0.004 | 0.996 |
|  | **Nickel (Ni)** | | | |
| Intercept | 0.250 | 2.223 | 0.112 | 0.911 |
| Air pressure | 0.000 | 0.002 | 0.021 | 0.983 |
| Air temperature | -0.006 | 0.003 | -1.813 | 0.071 |
| Relative humidity | 0.003 | 0.002 | 1.639 | 0.102 |
| Wind speed | -0.012 | 0.014 | -0.848 | 0.397 |
|  | **Lead (Pb)** | | | |
| Intercept | -38.553 | 26.776 | -1.440 | 0.151 |
| Air pressure | 0.043 | 0.026 | 1.697 | 0.091 |
| Air temperature | -0.026 | 0.040 | -0.637 | 0.525 |
| Relative humidity | 0.044 | 0.025 | 1.788 | 0.075 |
| Wind speed | -0.286 | 0.171 | -1.670 | 0.096 |
|  | **Antimony (Sb)** | | | |
| Intercept | 8.954 | 4.791 | 1.869 | 0.063 |
| Air pressure | -0.006 | 0.005 | -1.378 | 0.169 |
| Air temperature | -0.024 | 0.007 | -3.199 | 0.002 |
| Relative humidity | -0.014 | 0.004 | -3.174 | 0.002 |
| Wind speed | -0.047 | 0.030 | -1.538 | 0.125 |
|  | **Vanadium (V)** | | | |
| Intercept | 0.543 | 0.928 | 0.585 | 0.559 |
| Air pressure | 0.000 | 0.001 | -0.448 | 0.655 |
| Air temperature | 0.009 | 0.001 | 6.185 | 0.000 |
| Relative humidity | 0.000 | 0.001 | 0.429 | 0.668 |
| Wind speed | -0.001 | 0.006 | -0.216 | 0.829 |

**Figure S2.** The hierarchical cluster analysis with Euclidean distance as the distance measure (y-axis) and the Ward method as the primary agglomeration method. The cluster analysis was performed for all trace elements. It shows 13 objects: Co and Cd are the most similar elements (object 1), followed by object 2 (Co + Cd + V), object 3 (Co + Cd + V + Ni), and object 4 (Mo + Sb). The lower panel presents details on agglomeration for the remaining objects.


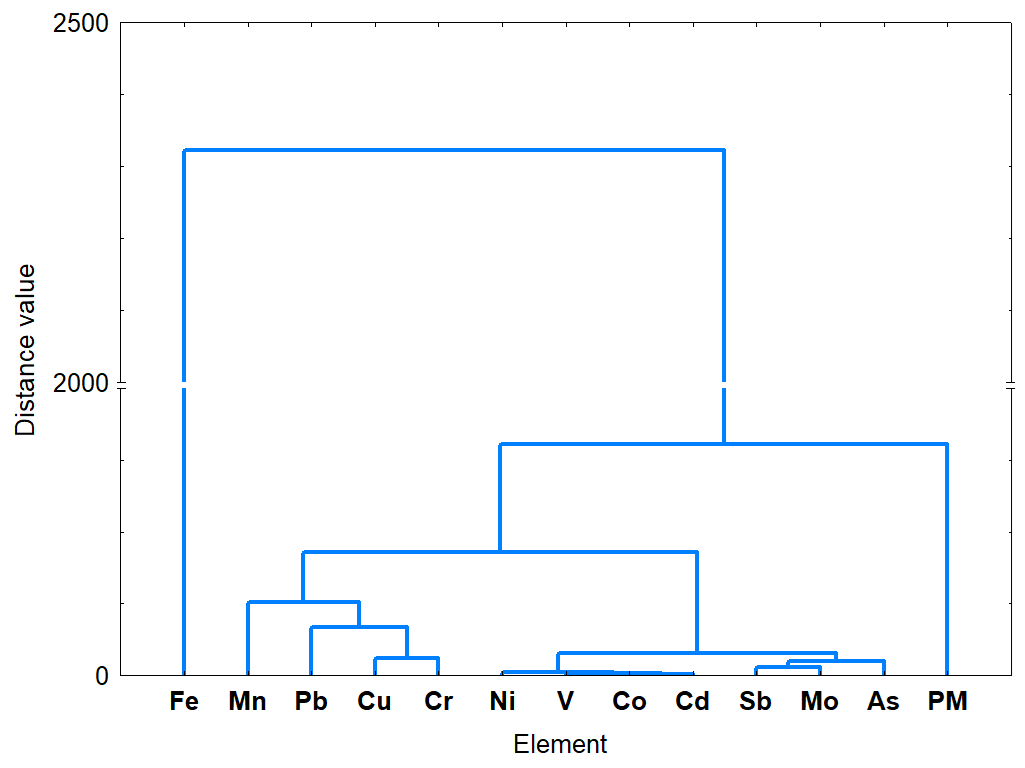


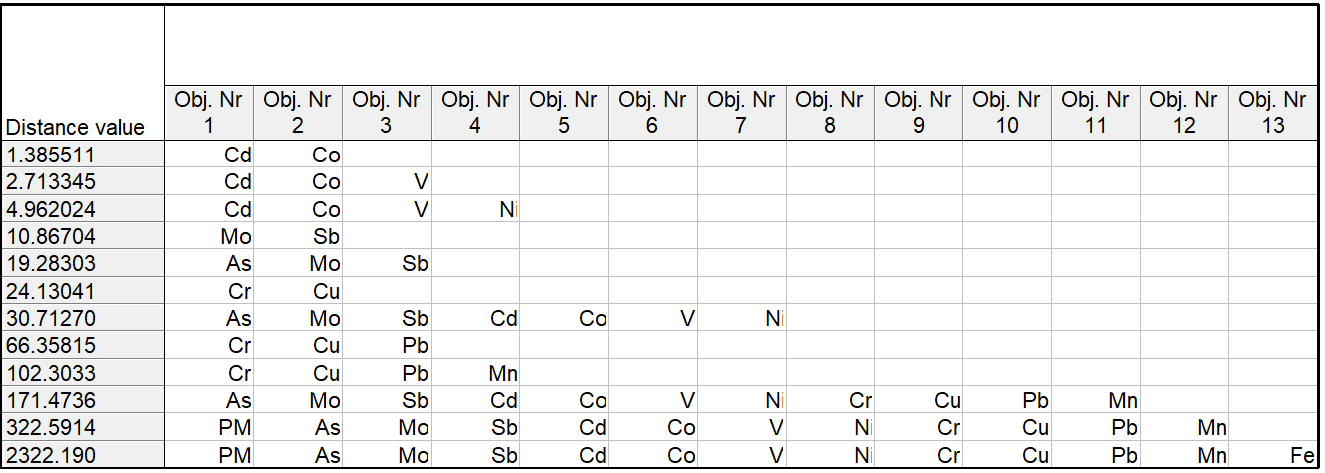


**Figure S3.** The scree plot of the number of principal components in PCA analysis. Eigenvalues > 1 are accepted.


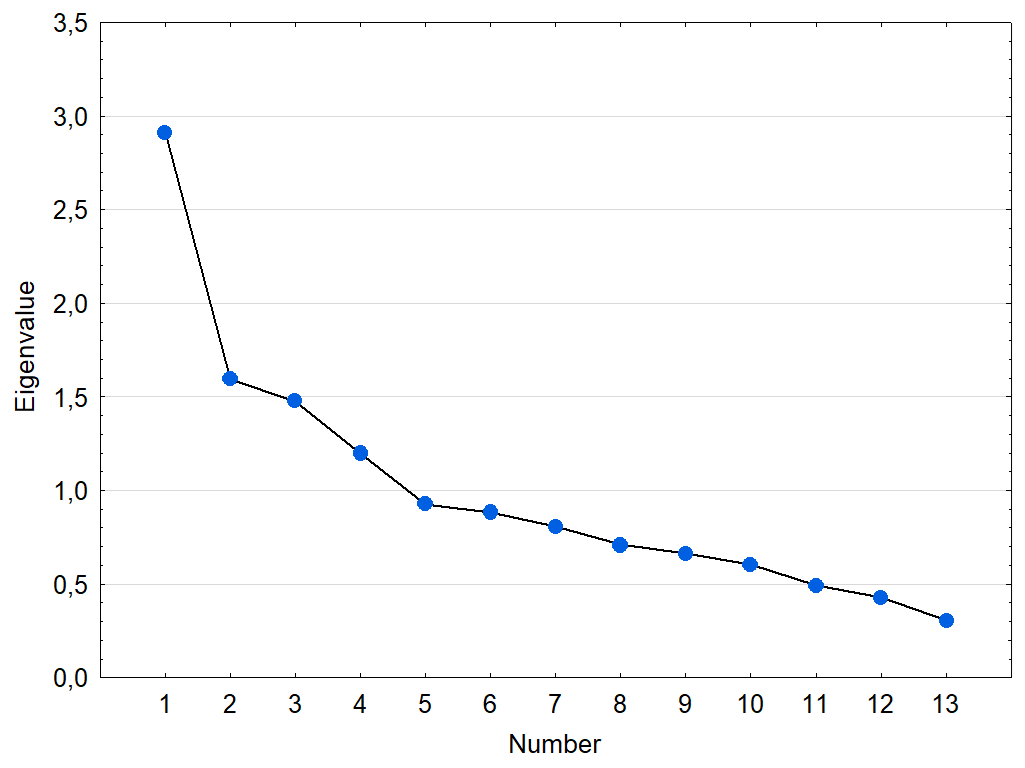

Supplement: Supplementary file 1 — Supplementary Material 1 [file 41598_2025_32170_MOESM1_ESM.docx]
